# Supplementary figures and images for: FGF signaling promotes spreading of fat body precursors necessary for adult adipogenesis in Drosophila
Source: PLoS Biol. 2023 Mar 22;21(3):e3002050. doi: 10.1371/journal.pbio.3002050 (PMC10069774; doi:10.1371/journal.pbio.3002050)

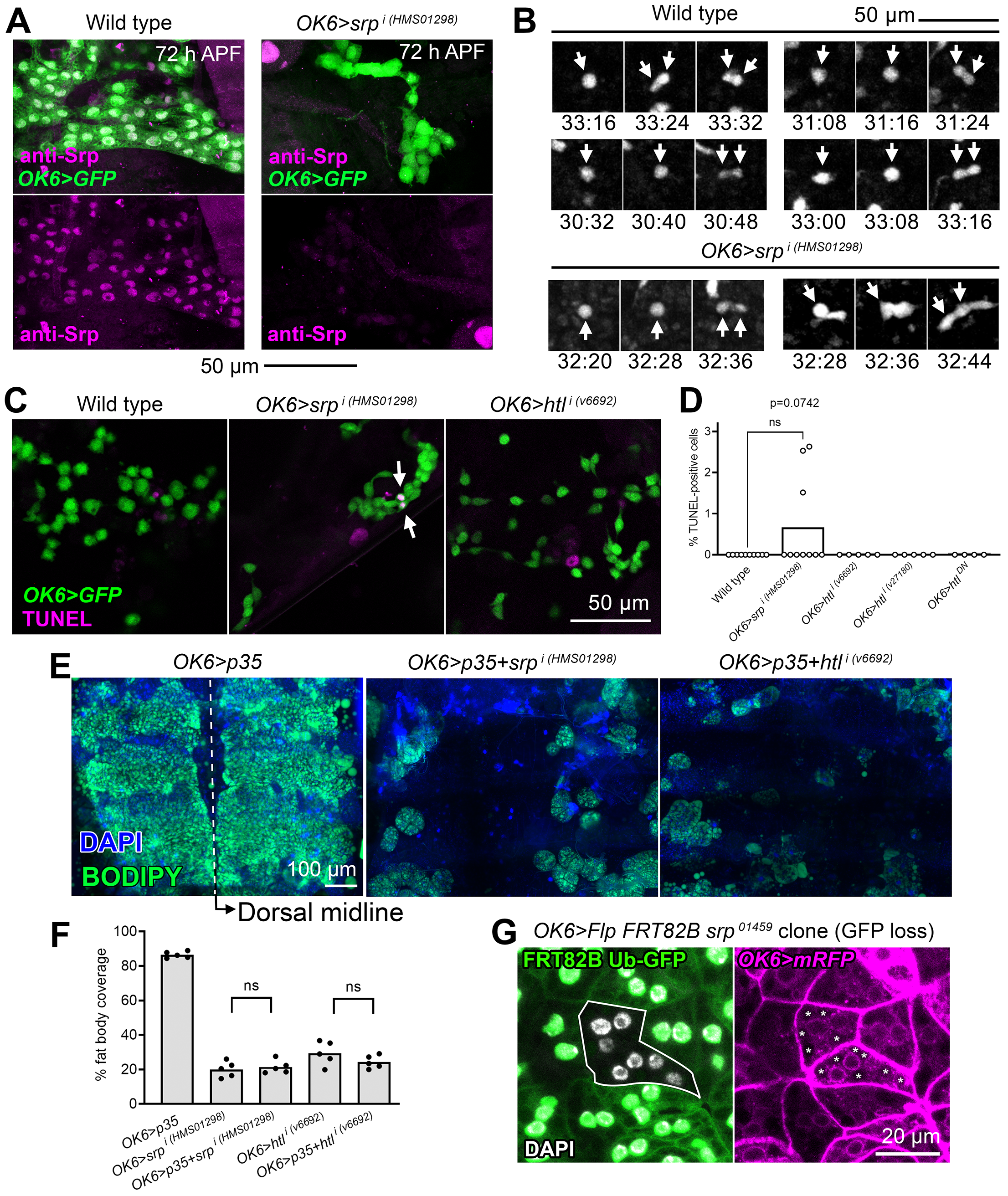

Supplement: S1 Fig — (A) Adult fat body precursors (OK6-GAL4-driven GFP, green) in wild-type control (left) and OK6>srpi (right) abdomens dissected 72 h APF and stained with anti-Srp antibody (magenta). (B) Still images from movies recorded between 30 and 36 h APF showing examples of cell division in adult fat body precursors (OK6-GAL4-driven GFP, white) from wild-type control and OK6>srpi animals. See also S2 Video. (C) TUNEL apoptosis staining (magenta) of adult fat body precursors (OK6-GAL4-driven GFP, green) from wild type, OK6>srpi and OK6>htli abdomens dissected 36 h APF. Apoptotic precursors (arrowheads) were observed in 3 out of 10 OK6>srpi. (D) Quantification of TUNEL-positive apoptotic adult fat body precursors in abdomens dissected 36 h APF of the indicated genotypes. Each dot represents the percentage of TUNEL positive precursors in 1 individual. Between 40 and 241 precursors were scored per individual. (E) Adult abdomens from control, OK6>srpi and OK6>htli flies, all expressing apoptosis inhibitor p35 under OK6-GAL4 control. Tissues stained with DAPI (nuclei, blue) and BODIPY (neutral lipids, green). (F) Coverage of adult fat body measured in images like those in (E) in at least 5 individuals per genotype, with the height of the bar indicating mean value. p35 expression shows no significant effect on OK6>srpi and OK6>htli phenotypes (unpaired t tests; n.s.: p > 0.05). Data for OK6>srpi and OK6>htli from Figs 2C and 5E, respectively. (G) High magnification view of srp01549 homozygous adult adipocytes generated in wild-type animals through mitotic recombination (see Fig 3). Mutant cells are negatively labeled by loss of Ub-GFP (green). OK6-GAL4-driven myr-RFP in magenta. Nuclei stained with DAPI (white). Asterisks mark circular spaces in the cytoplasm indicative of lipid droplets. The data underlying the graphs in the figure can be found in S1 Data. (TIF) [file pbio.3002050.s001.tif]

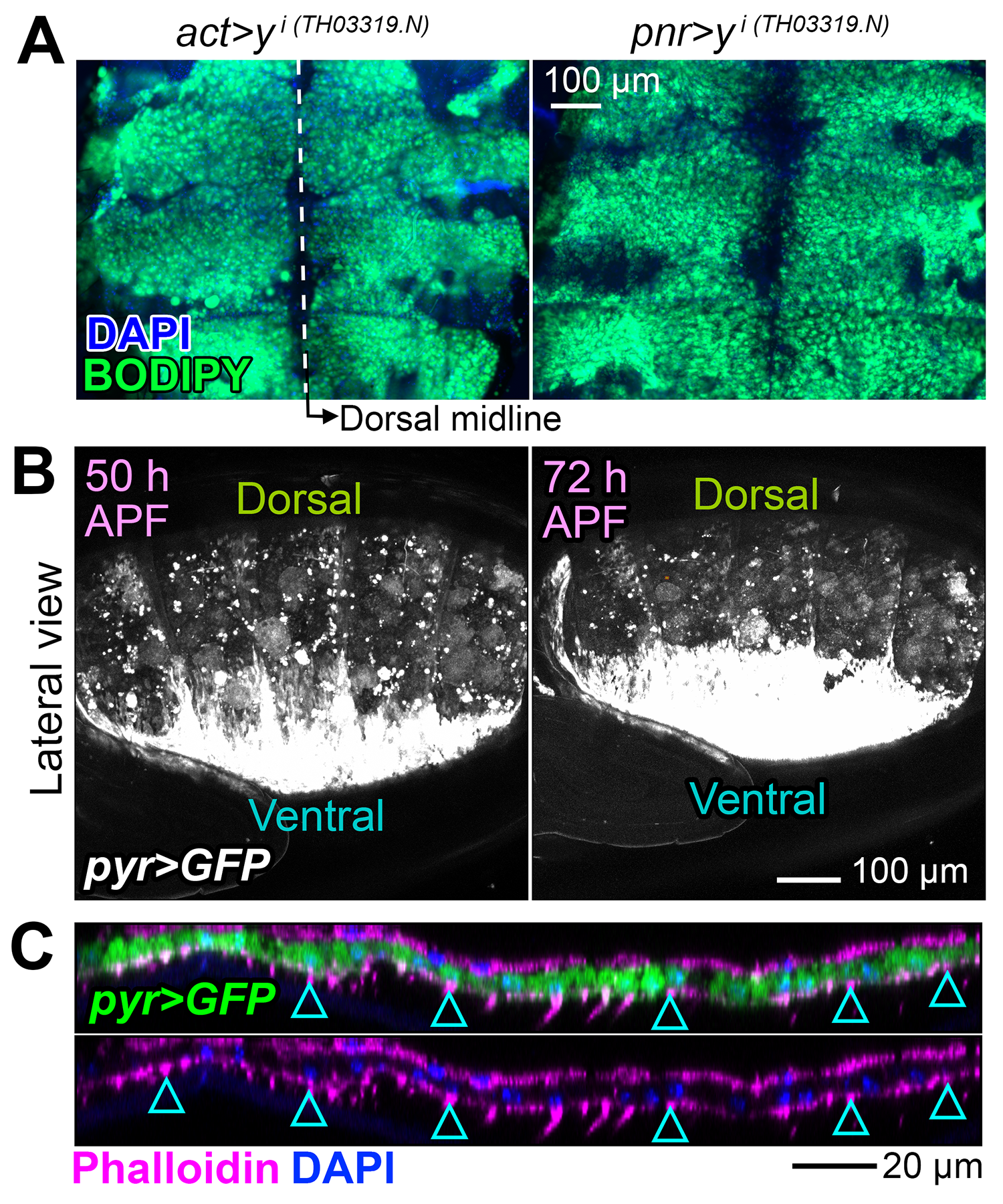

Supplement: S2 Fig — (A) Adult abdomens from control flies in which yellow was knocked down under control of act-GAL4 (act>yi) and pnr-GAL4 (pnr>yi). DAPI (blue) and BODIPY (green) stainings are shown. (B) Expression of GFP (white) under control of pyr-GAL4 in the ventral epidermis of the abdomen (lateral view) at 50 and 72 h APF. Images are maximum intensity projections of 60 confocal sections. (C) Z-section of an abdomen expressing GFP (green) under control of pyr-GAL4, dissected 72 h APF and stained with F-actin dye phalloidin (magenta). GFP-positive cells are the outermost cells and display actin-rich apical trichomes (arrowheads). Nuclei stained with DAPI (blue). (TIF) [file pbio.3002050.s002.tif]

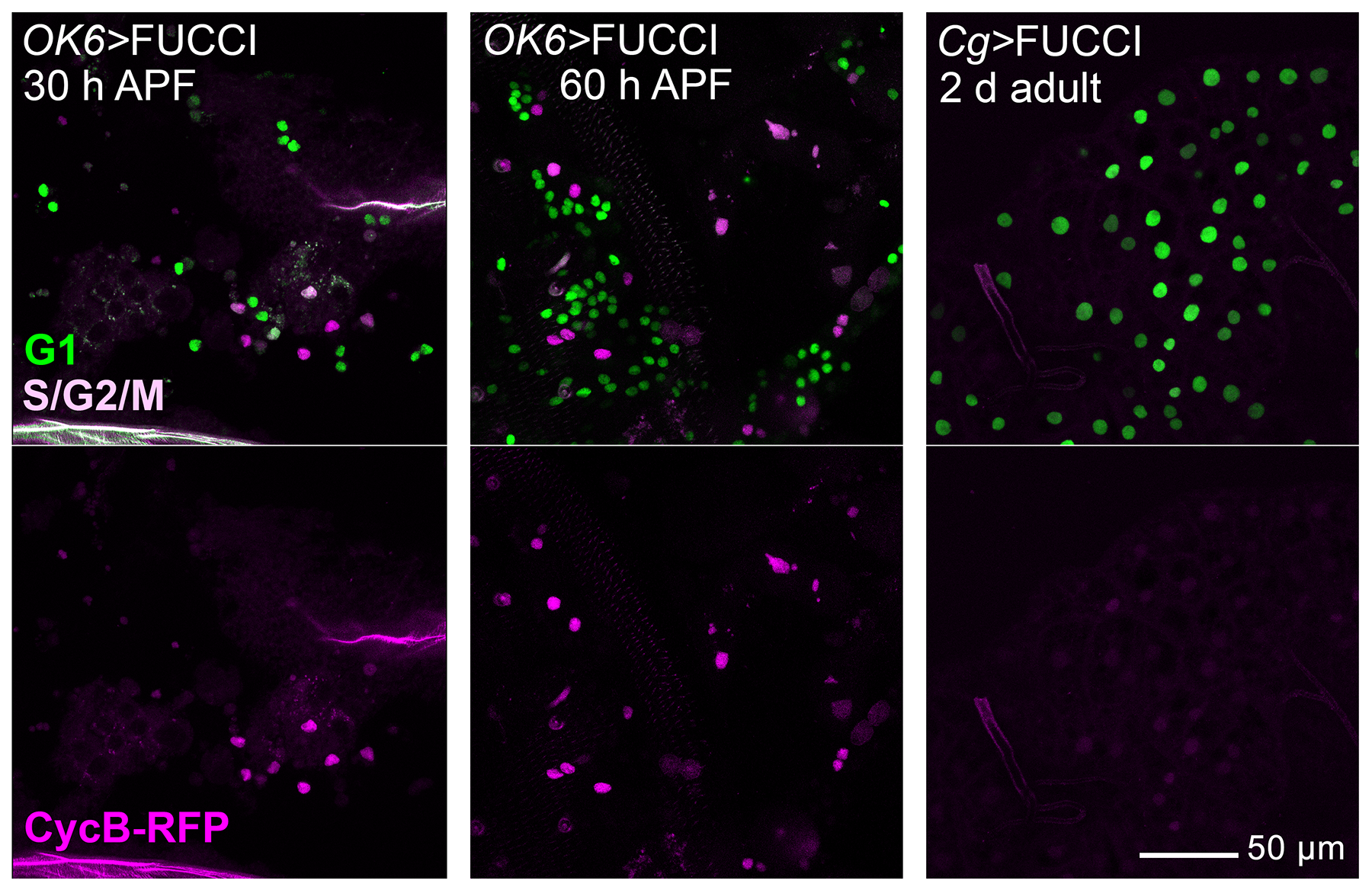

Supplement: S3 Fig — Adult fat body precursors (30 h APF and 60 h APF) and mature adipocytes (2 days after eclosion) expressing FUCCI system of cell cycle monitoring components [33] E2F-GFP (green) and CycB-RFP (magenta) under control of OK6-GAL4 (precursors) and Cg-GAL4 (mature adipocytes). In the adult, mature adipocytes are all found in G1 (accumulation of E2F-GFP in absence of CycB-RFP). CycB-RFP separately shown below. (TIF) [file pbio.3002050.s003.tif]
